# Medicinal Cannabis—Potential Drug Interactions

**DOI:** 10.3390/medicines6010003

**Published:** 2018-12-23

**Authors:** Muhammad A. Alsherbiny, Chun Guang Li

**Affiliations:** 1 NICM Health Research Institute, Western Sydney University, Westmead, NSW 2145, Australia; 2 Department of Pharmacognosy, Faculty of Pharmacy, Cairo University, Cairo 11562, Egypt

**Keywords:** Cannabis, cannabinoids, THC, CBD, drug–drug interactions, pharmacokinetic, cytochrome P450, UDP-glucuronosyltransferases, glycoprotein p, BCRP, MRPs

## Abstract

The endocannabinoids system (ECS) has garnered considerable interest as a potential therapeutic target in various carcinomas and cancer-related conditions alongside neurodegenerative diseases. Cannabinoids are implemented in several physiological processes such as appetite stimulation, energy balance, pain modulation and the control of chemotherapy-induced nausea and vomiting (CINV). However, pharmacokinetics and pharmacodynamics interactions could be perceived in drug combinations, so in this short review we tried to shed light on the potential drug interactions of medicinal cannabis. Hitherto, few data have been provided to the healthcare practitioners about the drug–drug interactions of cannabinoids with other prescription medications. In general, cannabinoids are usually well tolerated, but bidirectional effects may be expected with concomitant administered agents via affected membrane transporters (Glycoprotein p, breast cancer resistance proteins, and multidrug resistance proteins) and metabolizing enzymes (Cytochrome P450 and UDP-glucuronosyltransferases). Caution should be undertaken to closely monitor the responses of cannabis users with certain drugs to guard their safety, especially for the elderly and people with chronic diseases or kidney and liver conditions.

## 1. Introduction

**Figure 1:**
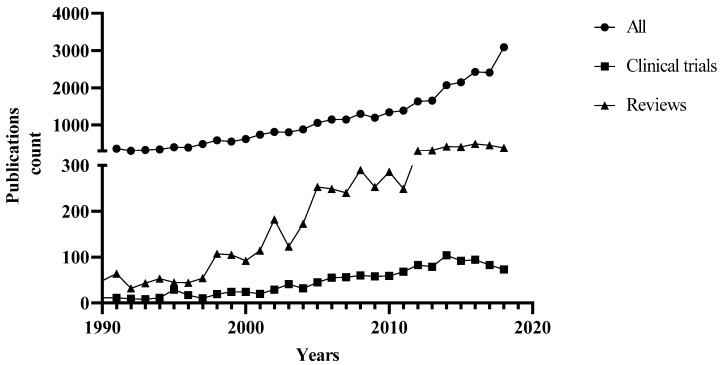
The PubMed retrieved publications (1990–2018) for studies on medicinal cannabis/marijuana/cannabinoids/tetrahydrocannabinol/cannabidiol.

The *Cannabis sativa* L. (cannabis) has long been used in traditional medicines around the world for treating various conditions [1]. Cannabis is used for either medicinal or recreational purposes, which are utterly based on the content of a group of compounds in the plant, designated as cannabinoids. Recently, there has been increasing interest in cannabis, as shown in the inclined publications, reviews, and clinical trials throughout the years (Figure 1), largely due to a change of attitudes towards the use of cannabis in many countries. For example, the FDA recently approved the first cannabis-derived drug (Epidiolex^®^) for the treatment of severe seizure disorders, and the projected sales of cannabidiol (CBD) products are estimated as high as $1.9 billion by 2020 [2].

It has been widely accepted that delta 9-tetrahydrocannabinol (Δ^9^-THC) alongside with less abundant Δ^8^-THC are the most potent psychoactive cannabinoids in cannabis. In contrast, cannabinol (CBN) and CBD lack the psychoactive properties. CBD can also interact with other receptors such as peroxisome proliferator-activated receptors (PPARs), orphan G-protein coupled receptor (GPR55), and transient receptor potential channel subfamily V member 1 (TRPV1) [3,4]. These receptors have been postulated as endocannabinoid receptors with debatable contributions in the endocannabinoid signalling [5]. The phytocannabinoids have been shown to have a range of biological activities by mimicking the endocannabinoids, for example, anandamide and 2-arachidonoylglycerol act as the endogenous ligand of cannabinoid receptors CB_1_ and CB_2_ [5,6,7]. The endocannabinoid system (ECS) as a potential therapeutic target for various pathological conditions has attracted a substantial interest, particularly in cancer treatment and neurological disorders [8]. In fact, inclined endocannabinoid level by either externally administered cannabinoids or by curtailing the degradation pathways might represent a useful strategy for developing new treatments for neurodegenerative diseases, nausea and vomiting, chronic pain, and several carcinomas. A recent review scrutinised the preclinical and clinical studies for the medical use of cannabis [8].

Hitherto, scattered bidirectional data on the effect of cannabinoids on mental health could be retrieved upon peri-pubertal exposure. For instance, the peri-pubertal administration of CBD prevented the behavioural abnormalities in schizophrenia animal models [9]. There is also preclinical evidence for the impaired extinction fear in adulthood for mice exposed to THC and stress concurrently in peri-adolescence; however, no effect was observed in animals exposed to either THC or stress alone [10]. Further clinical studies are warranted to confirm the long-term anxiety disorders and pathological fear in adulthood upon concomitant exposure to cannabis and stress by teenagers [10]. Furthermore, neurocognitive deficits with poorer psychomotor speed and working memory were reported in adolescents with high rates of cannabis use, but these effects were ameliorated effectively and affordably by aerobic fitness [11].

There are several reviews that have outlined the potentiality of cannabinoids as anticancer agents, alleviators of chemotherapy-induced nausea and vomiting (CINV), and cancer-related pain [8,12,13,14,15,16]. Studies of oral or oromucosal cannabinoid spray or pulmonary administration of cannabis smoke in oncology patients showed its tolerability with dose-dependent adverse effects (Table 1). Generally, cannabinoids containing products are used socially in cancer patients for its orexigenic, analgesic, antitumor, anxiolytic, and antiemetic effects [17]. The resiliency and complexity of cancer cells could rationalise the intervention with synergistic drug combinations, where smaller doses and curtailed side effects could be achieved. A cocktail of medications is usually given to cancer patients to overcome resilient cancer complexity, in most cases with combinatorial chemotherapeutic agents alongside alleviating medications such as antiemetics, appetite stimulant and pain killers. In this regard, there is potential to reduce or minimize the adverse effects of chemotherapeutic agents by using natural products such as cannabinoids (Table 1), which have been shown to have certain alleviating polypharmacological activities [18,19,20,21,22]. However, it is necessary to study the potential drug interactions, since there is still a lack of sufficient data out for clinical studies on possible interactions between cannabis and other prescription medications such as chemotherapeutic agents.

## 2. Potential Drug Interactions

Drug interactions can occur when two or more drugs/substances with similar or different actions (including herbal substances) are co-administrated, such as warfarin with aspirin, and cyclosporine A with St John’s Wort. Drug interactions may result from chemical reactions between different components or modifications by certain components of certain biochemical pathways involved in the action or metabolism of related drugs [33]. Drug interactions can be affected by various factors including disease and patient conditions, as well as the nature of the compounds involved. The potential outcome of a drug interaction can be additive (1 + 1 = 2), synergistic (1 + 1 > 2), or antagonistic (1 + 1 < 2). Therefore, a drug interaction may lead to an enhanced drug response or modified or unexpected adverse reactions. For example, a recent review advised patients receiving warfarin against concomitant cannabis use due to the probable risk of bleeding [34].

Generally speaking, drug–drug interactions are mediated by pharmacodynamic and/or pharmacokinetic mechanisms. On one hand, pharmacodynamic interactions comprise synergistic or antagonistic interactions on the same drug targets, e.g., receptors, which can often be anticipated and evaded. On the other hand, pharmacokinetic interactions involve alterations of the drug’s absorption, distribution, metabolism, and excretion (ADME). Most reported drug interactions are pharmacokinetic ones, e.g., through affecting drug metabolism enzymes such as cytochrome P450 (CYP450). CYP450 may be changed by interacting components through induction and inhibition. A longer period of time, for instance, several days is usually required for the induction of CYP450, which may lead to reduced drug plasma levels via increased metabolism, and consequently decreased drug effects. In contrast, the CYP450 inhibition is usually instantaneous and may lead to inclined drug plasma levels via enhanced metabolism, thus exaggerating the drug effects, which may result in substantial adverse reactions or toxicities [33]. Furthermore, cannabinoids bind to many members of membrane transporters e.g., ATP-binding cassette superfamily including breast cancer-resistant protein (BCRP) and Glycoprotein P (P-gp). Interactions of cannabinoids with BCRP [35,36] and P-gp [37,38,39] have been reported in preclinical studies. The duration of cannabinoids exposure affects the expression of P-gp [40,41] with downregulation in chronic exposure and upregulation in short exposure. Another family of transporters is multidrug resistance protein (MRP) which is coded by the ABCC gene and is involved in the transportation of various anticancer drugs [42]. An in-vitro study reported the modulation of expressions of MRP1, MRP2, MRP3, and MRP4 transporters by CB_1_ antagonists [43]. In addition, MRP1 transporter was inhibited differently by various cannabinoids, where CBD was the most potent inhibitor followed by CBN and THC, respectively [44]. Notably, the concentrations of cannabinoids used in these studies of the cannabinoid effects on membrane transporters are higher than that commonly measured in cannabis smokers [17]. 

Cannabis has been used in various forms as crude extracts or purified ingredients (with different THC/cannabinoids ratios); therefore, drug interactions caused by cannabis depend not only on the drugs involved but also the chemical components/profiles of the cannabis preparations used. 

## 3. Effects of Cannabis on Drug Metabolizing Enzymes and Related Drug Interactions

There are numerous in-vitro and in-vivo studies indicating that cannabinoids may act on P450 isoenzymes to affect the metabolism of various drugs. A systematic review by Stout & Cimino (2014) showed that P-450 is involved in metabolising several exogenous cannabinoids, for example tetrahydrocannabinol (THC; CYP2C9, 3A4), cannabidiol (CBD; CYPs 2C19, 3A4) and cannabinol (CBN; CYPs 2C9, 3A4), which is supported by clinical data on THC and CBD metabolism. The inhibition or induction of CYP by cannabinoids, e.g., THC as CYP 1A2 inducer and CBD as 3A4 inhibitor, may potentially affect the metabolism of many drugs metabolised by these CYPs. However, in many cases, the relevance of experimental findings in cells or animals to humans has yet to be established. Specific clinical studies are often needed to verify these interactions before a conclusion can be drawn. For example, studies showed that medicinal cannabis did not affect the clinical pharmacokinetics of irinotecan and docetaxel [45], while co-administration of cannabidiol (CBD) and clobazam (CLB) increased the blood CLB level in children with epilepsy [46]. A similar recent study showed that concomitant administration of CBD significantly changed serum levels of topiramate, rufinamide, clobazam, eslicarbazepine, and zonisamide in patients with treatment-resistant epilepsy [47]. Abnormal liver function test results were also noted in participants taking concomitant valproate, indicating the importance of monitoring serum levels of commonly used antiepileptic drugs and liver functions during treatment with CBD [47]. On the other hand, a study in healthy adults found that concomitant administration of fentanyl did not affect the plasma level of CBD, and the co-administration did not produce cardiovascular complications or respiratory depression during the test sessions and CBD did not potentiate fentanyl effects [48]; however, keloconazole (CYP3A4 inhibitor) was found to increase and rifampin (a CYP3A4 inducer) to reduce THC and CBD concentrations [49]. A cross-over study evaluated the use of cannabis tea, Bedrocan^®^ with chemotherapeutic agents, and reported no interactions with docetaxel and irinotecan [45].

A comprehensive overview of the pharmacokinetic interactions of synthetic and phytocannabinoids is summarised in Table 2.

## 4. Other Potential Drug Interactions

A study with 21 individuals showed that vaporized cannabis increased the analgesic effects of opioids without altering plasma opioid levels [32]. A non-controlled, prospective open-label study in 274 participants found that medicinal cannabis reduced the consumption of opioids [57]. The current research generally supports the use of medical cannabis as an adjunct or opioid substitute. On the other hand, it should be noted that a recent survey in the US indicates that cannabis may increase the risk of developing nonmedical prescription opioid use [58]. A study in 32 adult cannabis smokers found that low-dose alcohol (approximately 0.065% peak breath alcohol concentration) increased blood levels of THC, which may explain the performance impairment observed from a cannabis–alcohol combination [59,60]. Thus, it is important to develop a program at the state or national level to monitor the use of different forms of cannabis and their associations to different medical conditions. 

A study in a mouse neuropathic pain model found a synergistic interaction between gabapentin and THC, where gabapentin not only improved the THC therapeutic window, but also effectively enhanced its anti-allodynic activity [61].

In addition, there are early studies or case reports indicating potential drug interactions with warfarin, oxymorphone, pentobarbital, cocaine, sympathomimetic amines, disulfiram, disulfiram etc., but further research is needed. Interestingly, Russo (2016) mentioned that in extensive clinical application including complex drug regimens with opioids, tricyclic antidepressants, anticonvulsants etc, no drug interactions have been observed that would contraindicate or preclude the use of nabiximols with any specific pharmaceutical, although additive sedative effects are always possible [62]. MacCallum & Russo (2018) recently pointed out that there is no drug that cannot be used with cannabis, if necessary [63]. 

## 5. Conclusions

There is still limited data on significant drug interactions caused by medicinal cannabis. Thus, the evidence-based clinical guidelines on interactions of drugs with medicinal cannabis are still lacking. Nevertheless, caution should be undertaken to closely monitor the responses of cannabis users with certain drugs to guard their safety, especially for the elderly and people with chronic diseases or kidney and liver conditions.

## Figures and Tables

**Table 1:** Recent clinical studies of cannabinoids in oncology patients.

Cannabis Based Treatment	Study Type/Location/*n*	Dosage/Administration	Efficacy, Tolerability and Notes	References
**Chemotherapy Induced Nausea and Vomiting (CINV)**				
-Dronabinol [Marinol^®^; (-) trans Δ^9^-THC) alone or in combination with ondansetron (8–15 mg IV]	-Interventional (Placebo controlled). -*n* = 64. -USA.	-Capsule (2.5–20 mg). -Oral.	-Both were effective in CINV and well tolerated while dronabinol was more effective. -Combination is not more effective.	[23]
-Dronabinol [Marinol^®^; (-) trans Δ^9^-THC]	-Interventional (retrospective). -Children with malignancy.	-Solution administered orally (2.5–5 mg/m^2^ body surface every 6 h as needed).	-Positive response were reported for 60% of patients. -Prospective trial would be needed to confirm the dronabinol effect in CINV therapy.	[24]
-Nabilone with 5HT_3_ antagonist	-Interventional (retrospective) -*n* = 110 with median age 14 years with malignancy.	-Oral.	-Adverse effect was reported with minor clinical significance. -Poor nausea control in nabilone treated group.	[25]
**Cancer Pain**				
-Sativex^®^ (Δ^9^-THC: CBD at a ratio of 27:25 mg/mL) -THC (27 mg/mL)	-Interventional (Double Blind, Randomized, Parallel Group, Placebo Controlled), *n* = 177. -Phase 3. -UK.	-Oromucodal spray with maximum Δ^9^-THC: CBD (130:120 mg/day) or 130 mg/day Δ^9^-THC alone Each actuation is 100 μL.	-Compared with the placebo, the Sativex treated group showed significant pain relief unlike the Δ^9^-THC which was non-significant. -Reported adverse effects including dizziness, gastrointestinal disorders and confusion.	[26]
-Sativex^®^ (Δ^9^-THC: CBD at a ratio of 27:25 mg/mL)	-Interventional (single group assignment) -Phase 3. -UK.	-Oromucodal spray with maximum 130:120 mg/day of Δ^9^-THC: CBD.	-The long-term use is well tolerated without losing pain-relieving effects in terminal cancer-related pain refectory to opioids. -Adverse effects and tolerability assessed at the RCT withdrawal visit, 7–10 days later, then monthly, and at the withdrawal or completion of the study.	[27]
- Sativex^®^ (Δ^9^-THC: CBD at a ratio of 27:25 mg/mL)	-Interventional (Double Blind, Randomized, Parallel Group, Placebo Controlled). -Phase 3. -Multicentric. -*n* = 399.	-Oromucodal spray (100 μL per actuation twice daily in the morning and evening with a maximum of 10 sprays for 5 weeks).	-No significant difference was reported in advanced cancer patients with chronic pain (unalleviated with opioids). -Nabiximol still beneficial to secondary endpoints. -No evidence of abuse or misuse was reported.	[28]
			-No significant difference was reported in advanced cancer patients with chronic uncontrolled pain.	[29]
-Nabiximols (Sativex^®^; Δ^9^-THC: CBD at a ratio of 27:25 mg/mL)	-Interventional (Double Blind, Randomized, Parallel Group, Placebo Controlled). -Phase 2. -USA. -*n* = 360.	-Oromucodal spray in low (1–4 sprays/day), medium (6–10 sprays/day) and high (11–16 sprays/day) doses.	-Efficacy and safety were reported at low and medium doses against advanced cancer pain. -The adverse effects at high doses.	[30]
-Nabiximols (Sativex^®^, Δ^9^-THC: CBD at a ratio of 27:25 mg/mL)	-Interventional (Double-Blind, Placebo controlled, Crossover Pilot trial). -*n* = 16.	-Sublingual spray (7.5–30 mg/day).	-No significant difference was reported against chemotherapy-induced neuropathy. -Two-fold reduction of the pain in the responder group with adverse effects.	[31]
Cannabis cigarettes (3.56% Δ^9^-THC) in combination with opiates	-Interventional (open label).	-Pulmonary administration for chronic pain, including cancer patients.	-Declined chronic pain around 27% in patients receiving oxycodone or morphine analgesics. -No serious adverse effects were reported.	[32]

5HT_3_; 5-hydroxytryptamine 3 receptors, Δ^9^-THC; Delta -9 tetrahydrocannabinol, CBD; Cannabidiol, CINV; Chemotherapy induced Nausea and Vomiting, IV; Intravenous, *n*; number of participants, RCT; Randomised controlled trial.

**Table 2:** Overview of the recent reviews of the drug–drug interactions with cannabinoids.

Cannabinoid Based Treatment and Interactions	Affected Transporters and/or Metabolic Enzymes	Experimental Results, Notes and Outcomes	References
Cannabis, THC, CBD, CBN with either chemotherapies, abuse drugs or medications	-Membrane transporters ABC super family (glycoprotein P; P-gp, Breast cancer-resistance protein; BCRP, and multidrug resistance protein; MRP1, 2, 3 and 4) -Cytochrome P450 (3A, 2D6, 2C9, 1A1, 1A2, 1B1, 2B6 and 2C8) -UDP-glucuronosyltransferases (UGTs)	-**P-gp, BCRP, and MRP1-4** transporters expression were dysregulated by cannabinoids, but in higher concentrations than that usually measured in cannabis smokers. -**CYP3A** was competitively inhibited by THC, CBD and CBN, with CBD being the most potent in a concentration compatible with that in usual cannabis inhalation. -**CYP2D6** was inhibited by THC, CBD and CBN, with CBD being the most potent in a higher concentration than that in usual cannabis consumption. -**CYP2C9** was inhibited by THC, CBD and CBN, with CBD inhibitory effect being dependent on the used substrates. -**CYP1A1**, **1A2**, **1B1**, **2B6**, **2C19**, **3A4** and **2C8** were strongly inhibited by CBD. -**UGT1A9**, and **2B7** were inhibited by CBD. -**UGT1A7**, **1A8**, and **1A9** were inhibited by CBN. -**UGT2B7** was activated by CBN. Cannabinoids and drugs with inhibitory or stimulatory effects on UGT2B7 will interact. Clinical studies are warranted to explore the potential interactions with chemotherapy, alcohol, abuse drugs, and prescription medications.	[17,50,51]
Δ^9^-THC, CBD and marijuana inhalation with psychotropic agents	-Cytochrome P450	-**CYP2C9** and **CYP3A4** were inhibited by Δ^9^-THC. -**CYP2C19** and **CYP3A4** were inhibited by CBD. -**CYP1A1** and **CYP1A2** were induced by marijuana inhalation. Cannabinoids consumption via pyrolysis induced CYP due to aromatic hydrocarbons. The effect of cannabinoids on the CYP activity influenced by the formulation, administration route, and derivation (Plant based or synthetic). Clinical studies are warranted to explore the potential drug–drug interactions with cannabinoids.	[52]
Cannabinoids on other drugs	Cytochrome P450	-**CYP3A4** inhibitors and stimulators affect the elimination of Δ^9^-THC and CBD. Reviewed the pharmacokinetic interactions between cannabinoids on other drugs. Limited data on the drug’s effects on the accumulation of cannabinoids and marijuana. More clinical studies are warranted.	[53]
CBD with antiepileptic drugs	Cytochrome P450 or unknown	**Clinical studies of DDI**: -Non-significant increase of the clobazam plasma level administered with CBD (*n* = 13 children) due to potent inhibition of **CYP2C19**. -Significant change of plasma level of N-desmethylclobazam by CBD co-administration while no significant change in the level of valproate, stiripentol and levetiracetam (*n* = 24 open label trial). -All patients showed significant changes of the plasma levels of clobazam, N-desmethylclobazam, rufinamide, and topiramate by increasing CBD doses. The mean therapeutic range was exceeded for clobazam and N-desmethylclobazam; the plasma levels of eslicarbazepine and zonisamide were increased in adults only (*n* = 39 adults and 42 children). The purified CBD formula is FDA approved with antiepileptic drugs as a result of the published randomized clinical trials. CBD is well tolerated with potential DDI and adverse effects. The compulsory monitoring drug levels and patients’ liver functions are advised.	[47,54]
Synthetic and Phyto-cannabinoids	-Cytochrome P450 -UGTs	-**CYP1A** catalysed MROD activity was weakly inhibited by MAM-2201, JWH-019, STS-135, and UR-144. -**CYP2C8** catalysed amodiaquine N-deethylase was strongly inhibited by AM-2201, MAM-2201, and EAM-2201. -**CYP2C9** catalysed diclofenac hydroxylation and **CYP3A**-catalyzed midazolam 1′-hydroxylation were inhibited by AM-2201 and MAM-2201. -**CYP2C9** catalysed diclofenac 4′-hydroxylation, **CYP2C19**-catalyzed [S] -mephenytoin 4′-hydroxylation, and **CYP3A**-catalyzed midazolam 1′ hydroxylation were strongly inhibited by EAM-2201 (time-dependent inhibition). -**CYP2B6** and **CYP2C9** were strongly inhibited by THC, CBN and CBD. -**CYP2A6** was inhibited by THC and CBN (mechanism-based inhibition). -**CYP2D6** was competitively inhibited by CBD. -**CYP1A1 mRNA** expression was increased by THC in Hepa-1 cells, but EROD activity in CYP1A1 supersomes was inhibited by THC. -**CYP1A1**, **CYP1A2**, and **CYP1B1** were strongly inhibited by CBD (mechanism-based inhibition). -**CYP3A** was inhibited by CBD in human liver microsomes. -**CYP2C19**-catalyzed [S] -mephenytoin hydroxylation was inhibited by (CBD and THC (Mixed-type inhibition). -**UGT1A9**- and **UGT2B7** catalysed ethanol glucuronidation were non-competitively inhibited by CBD, and unlike the inclined ethanol glucuronidation in human liver microsome by CBN (dose dependent). -**UGT1A3** catalysed chenodeoxycholic acid 24-acylglucuronidation was strongly competitively inhibited by AM-2201, MAM-2201, and EAM-2201. -**UGT2B7**-mediated naloxone 3β-D-glucuronidation was competitively inhibited by AM-2201. Clinical studies of pharmacokinetics mediated drug interactions of synthetic and phyto-cannabinoids with the CYP and UTGs substrates are warranted.	[55,56]

ABC; ATP-binding cassette, AM-2201, EAM-2201, MAM-2201, JWH-019, STS-135, and UR-144; Synthetic cannabinoids, BCRP; Breast cancer resistance proteins, CBD; Cannabidiol, CBN; cannabinol, CYP; Cytochrome P450, DDI; drug–drug interactions, MROD; 7-methoxyresorufin O-demethylation, MRP; Multidrug resistance proteins, P-gp; Glycoprotein P, THC; tetrahydrocannabinol, UGTs; UDP-glucuronosyltransferases.

## References

[1] Bonini S.A., Premoli M., Tambaro S., Kumar A., Maccarinelli G., Memo M., Mastinu A. (2018). Cannabis sativa: A comprehensive ethnopharmacological review of a medicinal plant with a long history. J. Ethnopharmacol..

[2] Corroon J., Kight R. (2018). Regulatory Status of Cannabidiol in the United States: A Perspective. Cannabis Cannabinoid Res..

[3] Brown A. (2007). Novel cannabinoid receptors. Br. J. Pharmacol..

[4] De Petrocellis L., Di Marzo V. (2010). Non-CB 1, non-CB 2 receptors for endocannabinoids, plant cannabinoids, and synthetic cannabimimetics: Focus on G-protein-coupled receptors and transient receptor potential channels. J. Neuroimmune Pharmacol..

[5] Pertwee R.G., Howlett A., Abood M.E., Alexander S., Di Marzo V., Elphick M., Greasley P., Hansen H.S., Kunos G., Mackie K. (2010). International Union of Basic and Clinical Pharmacology. LXXIX. Cannabinoid receptors and their ligands: Beyond CB1 and CB2. Pharmacol. Rev..

[6] Matsuda L.A., Lolait S.J., Brownstein M.J., Young A.C., Bonner T.I. (1990). Structure of a cannabinoid receptor and functional expression of the cloned cDNA. Nature.

[7] Munro S., Thomas K.L., Abu-Shaar M. (1993). Molecular characterization of a peripheral receptor for cannabinoids. Nature.

[8] Fraguas-Sánchez A.I., Torres-Suárez A.I. (2018). Medical Use of Cannabinoids. Drugs.

[9] Peres F.F., Diana M.C., Levin R., Suiama M.A., Almeida V., Vendramini A.M., Santos C.M., Zuardi A.W., Hallak J.E., Crippa J.A. (2018). Cannabidiol administered during peri-adolescence prevents behavioral abnormalities in an animal model of schizophrenia. Front. Pharmacol..

[10] Saravia R., Ten-Blanco M., Julià-Hernández M., Gagliano H., Andero R., Armario A., Maldonado R., Berrendero F. (2018). Concomitant THC and stress adolescent exposure induces impaired fear extinction and related neurobiological changes in adulthood. Neuropharmacology.

[11] Wade N.E., Wallace A.L., Swartz A.M., Lisdahl K.M. (2018). Aerobic Fitness Level Moderates the Association Between Cannabis Use and Executive Functioning and Psychomotor Speed Following Abstinence in Adolescents and Young Adults. J. Int. Neuropsychol. Soc..

[12] Guzmán M. (2003). Cannabinoids: Potential anticancer agents. Nat. Rev. Cancer.

[13] Bogdanovic V., Mrdjanovic J., Borisev I. (2017). A Review of the Therapeutic Antitumor Potential of Cannabinoids. J. Altern. Compl. Med..

[14] Vuger A.T., Šeparović R., Silovski T., Pavlović M., Pavlica V., Knežević S.V. (2016). Cannabis in oncology. Libri Oncol..

[15] Davis M.P. (2016). Cannabinoids for Symptom Management and Cancer Therapy: The Evidence. J. Natl. Comprehen. Cancer Netw..

[16] Velasco G., Hernández-Tiedra S., Dávila D., Lorente M. (2016). The use of cannabinoids as anticancer agents. Prog. Neuro-Psychopharmacol. Biol. Psychiatry.

[17] Bouquié R., Deslandes G., Mazaré H., Cogné M., Mahé J., Grégoire M., Jolliet P. (2018). Cannabis and anticancer drugs: Societal usage and expected pharmacological interactions—A review. Fundam. Clin. Pharmacol..

[18] Xie L., Xie L., Kinnings S.L., Bourne P.E. (2012). Novel computational approaches to polypharmacology as a means to define responses to individual drugs. Annu. Rev. Pharmacol. Toxicol..

[19] Liu H., Wang J., Zhou W., Wang Y., Yang L. (2013). Systems approaches and polypharmacology for drug discovery from herbal medicines: An example using licorice. J. Ethnopharmacol..

[20] Alsherbiny M.A., Abd-Elsalam W.H., El badawy S.A., Taher E., Fares M., Torres A., Chang D., Guang Li C. (2018). Ameliorative and protective effects of ginger and its main constituents against natural, chemical and radiation-induced toxicities: A comprehensive review. Food Chem. Toxicol..

[21] Alsherbiny M.A., Ezzat S.M., Elsakhawy F.S., Kamel G.M., Abdel-Kawy M.A. (2015). Impact of certain Solanum speciess natural products as potent cytotoxic and anti-Inflammatory agents. J. Med. Plants Res..

[22] Ho T.T., Tran Q.T., Chai C.L. (2018). The polypharmacology of natural products. Futur. Med. Chem..

[23] Meiri E., Jhangiani H., Vredenburgh J.J., Barbato L.M., Carter F.J., Yang H.-M., Baranowski V. (2007). Efficacy of dronabinol alone and in combination with ondansetron versus ondansetron alone for delayed chemotherapy-induced nausea and vomiting. Curr. Med. Res. Opin..

[24] Elder J.J., Knoderer H.M. (2015). Characterization of dronabinol usage in a pediatric oncology population. J. Pediatr. Pharmacol. Ther..

[25] Polito S., MacDonald T., Romanick M., Jupp J., Wiernikowski J., Vennettilli A., Khanna M., Patel P., Ning W., Sung L. (2018). Safety and efficacy of nabilone for acute chemotherapy-induced vomiting prophylaxis in pediatric patients: A multicenter, retrospective review. Pediatr. Blood Cancer.

[26] Johnson J.R., Burnell-Nugent M., Lossignol D., Ganae-Motan E.D., Potts R., Fallon M.T. (2010). Multicenter, double-blind, randomized, placebo-controlled, parallel-group study of the efficacy, safety, and tolerability of THC:CBD extract and THC extract in patients with intractable cancer-related pain. J. Pain Symp. Manag..

[27] Fallon M.T., Albert Lux E., McQuade R., Rossetti S., Sanchez R., Sun W., Wright S., Lichtman A.H., Kornyeyeva E. (2017). Sativex oromucosal spray as adjunctive therapy in advanced cancer patients with chronic pain unalleviated by optimized opioid therapy: Two double-blind, randomized, placebo-controlled phase 3 studies. Br. J. Pain.

[28] Lichtman A.H., Lux E.A., McQuade R., Rossetti S., Sanchez R., Sun W., Wright S., Kornyeyeva E., Fallon M.T. (2018). Results of a Double-Blind, Randomized, Placebo-Controlled Study of Nabiximols Oromucosal Spray as an Adjunctive Therapy in Advanced Cancer Patients with Chronic Uncontrolled Pain. J. Pain Symp. Manag..

[29] Johnson J.R., Lossignol D., Burnell-Nugent M., Fallon M.T. (2013). An open-label extension study to investigate the long-term safety and tolerability of THC/CBD oromucosal spray and oromucosal THC spray in patients with terminal cancer-related pain refractory to strong opioid analgesics. J. Pain Symp. Manag..

[30] Portenoy R.K., Ganae-Motan E.D., Allende S., Yanagihara R., Shaiova L., Weinstein S., McQuade R., Wright S., Fallon M.T. (2012). Nabiximols for opioid-treated cancer patients with poorly-controlled chronic pain: A randomized, placebo-controlled, graded-dose trial. J. Pain Off. J. Am. Pain Soc..

[31] Lynch M.E., Cesar-Rittenberg P., Hohmann A.G. (2014). A Double-Blind, Placebo-Controlled, Crossover Pilot Trial With Extension Using an Oral Mucosal Cannabinoid Extract for Treatment of Chemotherapy-Induced Neuropathic Pain. J. Pain Symp. Manag..

[32] Abrams D.I., Couey P., Shade S.B., Kelly M.E., Benowitz N.L. (2011). Cannabinoid–Opioid Interaction in Chronic Pain. Clin. Pharmacol. Ther..

[33] Li C.G., Yang L., Zhou S.-F. (2007). Interactions between Chinese herbal medicines and drugs. Aust. J. Acupunct. Chin. Med..

[34] Damkier P., Lassen D., Christensen M.M.H., Madsen K.G., Hellfritzsch M., Pottegård A. (2018). Interaction between warfarin and cannabis. Basic Clin. Pharmacol. Toxicol..

[35] Holland M., Lau D., Allen J., Arnold J. (2007). The multidrug transporter ABCG2 (BCRP) is inhibited by plant-derived cannabinoids. Br. J. Pharmacol..

[36] Feinshtein V., Erez O., Ben-Zvi Z., Eshkoli T., Sheizaf B., Sheiner E., Holcberg G. (2013). Cannabidiol enhances xenobiotic permeability through the human placental barrier by direct inhibition of breast cancer resistance protein: An ex vivo study. Am. J. Obstet. Gynecol..

[37] Holland M., Panetta J., Hoskins J., Bebawy M., Roufogalis B., Allen J., Arnold J. (2006). The effects of cannabinoids on P-glycoprotein transport and expression in multidrug resistant cells. Biochem. Pharmacol..

[38] Zhu H.-J., Wang J.-S., Markowitz J.S., Donovan J.L., Gibson B.B., Gefroh H.A., DeVane C.L. (2006). Characterization of P-glycoprotein inhibition by major cannabinoids from marijuana. J. Pharmacol. Exp. Ther..

[39] Tournier N., Chevillard L., Megarbane B., Pirnay S., Scherrmann J.-M., Decleves X. (2010). Interaction of drugs of abuse and maintenance treatments with human P-glycoprotein (ABCB1) and breast cancer resistance protein (ABCG2). Int. J. Neuropsychopharmacol..

[40] Arnold J.C., Hone P., Holland M.L., Allen J.D. (2012). CB2 and TRPV1 receptors mediate cannabinoid actions on MDR1 expression in multidrug resistant cells. Pharmacol. Rep..

[41] Feinshtein V., Erez O., Ben-Zvi Z., Erez N., Eshkoli T., Sheizaf B., Sheiner E., Huleihel M., Holcberg G. (2013). Cannabidiol changes P-gp and BCRP expression in trophoblast cell lines. PeerJ.

[42] Marquez B., Van Bambeke F. (2011). ABC multidrug transporters: Target for modulation of drug pharmacokinetics and drug-drug interactions. Curr. Drug Targets.

[43] Wittgen H.G., van den Heuvel J.J., van den Broek P.H., Dinter-Heidorn H., Koenderink J.B., Russel F.G. (2011). Cannabinoid CB1 receptor antagonists modulate transport activity of multidrug resistance-associated proteins MRP1, MRP2, MRP3, and MRP4. Drug Metab. Dispos..

[44] Holland M.L., Allen J.D., Arnold J.C. (2008). Interaction of plant cannabinoids with the multidrug transporter ABCC1 (MRP1). Eur. J. Pharmacol..

[45] Engels F.K., De Jong F.A., Sparreboom A., Mathot R.A., Loos W.J., Kitzen J.J., De Bruijn P., Verweij J., Mathijssen R.H. (2007). Medicinal cannabis does not influence the clinical pharmacokinetics of irinotecan and docetaxel. Oncologist.

[46] Geffrey A.L., Pollack S.F., Bruno P.L., Thiele E.A. (2015). Drug–drug interaction between clobazam and cannabidiol in children with refractory epilepsy. Epilepsia.

[47] Gaston T.E., Bebin E.M., Cutter G.R., Liu Y., Szaflarski J.P., Program U.C. (2017). Interactions between cannabidiol and commonly used antiepileptic drugs. Epilepsia.

[48] Manini A.F., Yiannoulos G., Bergamaschi M.M., Hernandez S., Olmedo R., Barnes A.J., Winkel G., Sinha R., Jutras-Aswad D., Huestis M.A. (2015). Safety and pharmacokinetics of oral cannabidiol when administered concomitantly with intravenous fentanyl in humans. J. Addict. Med..

[49] Stout S.M., Cimino N.M. (2014). Exogenous cannabinoids as substrates, inhibitors, and inducers of human drug metabolizing enzymes: A systematic review. Drug Metab. Rev..

[50] Arellano A.L., Papaseit E., Romaguera A., Torrens M., Farre M. (2017). Neuropsychiatric and General Interactions of Natural and Synthetic Cannabinoids with Drugs of Abuse and Medicines. CNS Neurol. Disord. Drug Targets.

[51] Zendulka O., Dovrtelová G., Nosková K., Turjap M., Sulcová A., Hanus L., Jurica J. (2016). Cannabinoids and cytochrome P450 interactions. Curr. Drug Metab..

[52] Rong C., Carmona N.E., Lee Y.L., Ragguett R.M., Pan Z., Rosenblat J.D., Subramaniapillai M., Shekotikhina M., Almatham F., Alageel A. (2018). Drug-drug interactions as a result of co-administering Delta(9)-THC and CBD with other psychotropic agents. Expert Opin. Drug Saf..

[53] Anderson G.D., Chan L.N. (2016). Pharmacokinetic Drug Interactions with Tobacco, Cannabinoids and Smoking Cessation Products. Clin. Pharm..

[54] Gaston T.E., Szaflarski J.P. (2018). Cannabis for the Treatment of Epilepsy: An Update. Curr. Neurol. Neurosci. Rep..

[55] Kong T.Y., Kim J.H., Kim D.K., Lee H.S. (2018). Synthetic cannabinoids are substrates and inhibitors of multiple drug-metabolizing enzymes. Arch. Pharm. Res..

[56] Tai S., Fantegrossi W.E., Baumann M.H., Glennon R.A., Wiley J.L. (2017). Pharmacological and Toxicological Effects of Synthetic Cannabinoids and Their Metabolites. Neuropharmacology of New Psychoactive Substances (NPS): The Science Behind the Headlines.

[57] Haroutounian S., Ratz Y., Ginosar Y., Furmanov K., Saifi F., Meidan R., Davidson E. (2016). The effect of medicinal cannabis on pain and quality-of-life outcomes in chronic pain. Clin. J. Pain.

[58] Olfson M., Wall M.M., Liu S.-M., Blanco C. (2017). Cannabis use and risk of prescription opioid use disorder in the United States. Am. J. Psychiatry.

[59] Ronen A., Chassidim H.S., Gershon P., Parmet Y., Rabinovich A., Bar-Hamburger R., Cassuto Y., Shinar D. (2010). The effect of alcohol, THC and their combination on perceived effects, willingness to drive and performance of driving and non-driving tasks. Accid. Anal. Prev..

[60] Hartman R.L., Brown T.L., Milavetz G., Spurgin A., Gorelick D.A., Gaffney G., Huestis M.A. (2015). Controlled cannabis vaporizer administration: Blood and plasma cannabinoids with and without alcohol. Clin. Chem..

[61] Atwal N., Casey S.L., Mitchell V.A., Vaughan C.W. (2019). THC and gabapentin interactions in a mouse neuropathic pain model. Neuropharmacology.

[62] Russo E.B. (2016). Current therapeutic cannabis controversies and clinical trial design issues. Front. Pharmacol..

[63] MacCallum C.A., Russo E.B. (2018). Practical considerations in medical cannabis administration and dosing. Eur. J. Intern. Med..

